# Interdependent relationship between depression and Internet gaming disorder in parent-child dyads: The mediating role of family relationship and gaming time

**DOI:** 10.1371/journal.pone.0351947

**Published:** 2026-06-15

**Authors:** Qian Li, Yilun Huang, Samuel Yeung-Shan Wong, Winnie W. S. Mak, Xue Yang

**Affiliations:** 1 The Jockey Club School of Public Health and Primary Care, The Chinese University of Hong Kong, Hong Kong SAR; 2 Department of Psychology, The Chinese University of Hong Kong, Hong Kong SAR; Yale University, UNITED STATES OF AMERICA

## Abstract

**Background and objective:**

A well-established link exists between depression and Internet gaming disorder (IGD) at the individual level, while it remains unexplored within the family system. This study aims to investigate the interdependent relationship between parent and adolescent depression and IGD, and to identify the potential mechanisms.

**Methods:**

A cross-sectional dyadic study was conducted with adolescents and their parents (primary caregiver) in Hong Kong. Adolescents completed anonymous surveys in classrooms, and parents completed online surveys via WhatsApp or phone interviews. The Actor-Partner Interdependence Model (APIM) and Actor-Partner Interdependence Mediation Model (APIMeM) were utilized to test the interdependence and mediators between depression and IGD in parent-child dyads, respectively.

**Results:**

A total of 1,277 parent-child dyads were included. Depressive symptoms in parents (β = 0.072) and adolescents (β = 0.273, both p < 0.05) were positively associated with their own IGD symptoms (actor effect). Adolescent depressive symptoms were positively associated with parental IGD symptoms (β = 0.078, p < 0.05). Family relationships and adolescent gaming time mediated the association of adolescent depressive symptoms with adolescent IGD symptoms (indirect effect accounting for 21.5%) and parental IGD symptoms (74.3%).

**Conclusions:**

Adolescent depressive symptoms were positively associated with their own and parental IGD symptoms, which were mediated by adolescent-reported family relationships and adolescent gaming time. The influence of adolescents’ mental health problems on parents’ problematic behaviors within the family system should not be overlooked.

## Introduction

Internet Gaming Disorder (IGD) is defined as “a pattern of excessive and prolonged Internet gaming that leads to a cluster of cognitive and behavioral symptoms, including progressive loss of control over gaming, tolerance, and withdrawal symptoms, analogous to the symptoms of substance use disorders” in the Diagnostic and Statistical Manual of Mental Disorders, Fifth Edition (DSM-5) [1]. IGD has become a significant global public health concern, particularly in Asia. A meta-analysis [2] across 33 countries reported a global pooled prevalence of adolescent IGD of 8.8% in 2022. In China, the pooled prevalence of adolescent IGD in 2024 was 10% [3]. Furthermore, Internet gaming has seen a significant rise in popularity among middle-aged and older adults, including parents. A recent gamers report in Hong Kong [4] showed that over 60% of adults aged 35–54 years engaged in Internet games. In addition, a prior research reported that around half of mothers and over 60% of fathers in Hong Kong were Internet gamers [5]. Whereas the IGD among this age group, particularly in parents, received little attention. Previous studies solely reported the IGD prevalence either in young adults (pooled: 6.1%) [6] or in adults across broad age ranges (pooled: 1.9%) [7]. No studies have reported it in middle-aged adults or parents.

### The relationship between depression and IGD at the individual level

Depression may increase vulnerability to IGD as individuals may engage in excessive gaming to escape negative emotions, cope with distress [8], or compensate for a lack of reward in their offline lives [9], thereby reinforcing compulsive use patterns. While the predictive role of depression on IGD has been well-established at the individual level among adolescents and young adults [10], a significant gap exists in understanding this dynamic within the family system and middle-aged adults—particularly parents. Parents not only face unique stressors related to work and family responsibilities [11,12] but also exert a profound influence on their children’s mental and behavioral health [13]. For instance, a cross-sectional study [14] found that individuals with IGD reported significantly higher depressive symptoms, yet the mean age of participants was 29 years, leaving the experience of older adults underexplored. Crucially, no studies have specifically examined the association between depression and IGD in parents.

### The Parent-Adolescent Link in Depression and IGD

Parents play a critical role in adolescents’ behavioral development [13], including their engagement with digital media. Research indicates that parental affective disorders can increase adolescents’ vulnerability to various digital addictions [15]. While the association between parental depression and adolescents’ IGD has received increasing attention, findings remain inconsistent. Our literature review (**S1 File** **and** **S1 Table**) identified six studies examining this relationship in parent-child dyads. Three studies (one longitudinal and two cross-sectional, with N ranging from 104 to 4,835 dyads) reported that parental depression was positively associated with adolescent IGD, either directly or indirectly through poorer parent-child relationships. In contrast, one cross-sectional study from Germany (N = 1,095 dyads) found an inverse association, while two other longitudinal studies reported no significant link. The Family Systems Theory [16] indicates that parent-child interactions are bidirectional and that the emotions and behaviors of family members can mutually influence each other within the family system. Notably, the reverse pathway-how adolescent depression may influence parental IGD-has been entirely overlooked. This is a critical gap, given evidence that children’s mental health problems can increase parental stress [17]. According to the General Strain Theory [18], individuals under significant stress may adopt maladaptive coping strategies. Thus, parents of depressed adolescents may turn to excessive gaming as an emotional escape, potentially elevating their own risk of IGD. Examining these bidirectional, interdependent relationships within the family system is essential for a complete understanding of how depression and IGD are mutually reinforced across generations.

### Mediating roles of family relationships and gaming time

Family relationships and gaming time are two critical pathways through which depression and IGD may be linked within parent-child dyads. First, poor family relationship was found to mediate the associations between parental depression and adolescent problematic gaming in a cross-sectional study [19]. The Family Systems Theory [16] posits that emotional disturbances in one member affect the entire family unit. The Emotional Security Theory [20] further explains this pathway, as parental depressive symptoms can undermine the family’s emotional security, precipitating maladaptive coping behaviors in adolescents [21]. Conversely, adolescent depression can also disrupt family dynamics by increasing conflict and reducing communication [22], potentially elevating parental stress. According to the General Strain Theory [18], such heightened stress may lead parents to adopt maladaptive coping strategies, such as excessive gaming. Despite these plausible bidirectional pathways, no study has tested the mediating role of family relationships using a dyadic framework like the Actor-Partner Interdependence Mediation Model (APIMeM).

Second, gaming time itself may serve as a direct behavioral pathway. It is a well-established risk factor for IGD in both adolescents [23] and adults [24]. Parental depression may influence this pathway; for instance, parental psychological problems have been linked to increased adolescent screen time, including gaming [25]. Similarly, following the General Strain Theory [18], the stress resulting from a child’s depression could lead parents to increase their own gaming time as a coping mechanism. However, the potential for adolescent and parental gaming time to mediate the depression-IGD link reciprocally within dyads remains unexplored in the literature.

### The current study

Building on the identified gaps, this study addresses two critical limitations in the existing literature. First, prior research has predominantly adopted a unidirectional perspective, focusing on how parental depression influences adolescent IGD while neglecting the potential reciprocal effects of adolescent well-being on parental mental health and behavior. Second, the underlying mechanisms remain unexplored. To bridge these gaps, the present study aims: 1) to examine the bidirectional, interdependent relationships between depression and IGD in parent-adolescent dyads using the Actor-Partner Interdependence Model (APIM) within a Hong Kong dyadic sample; and 2) to investigate whether family relationship quality and both adolescent and parental gaming time mediate these dyadic associations.

## Methods

### Study design and participants

This dyadic cross-sectional study was conducted in Hong Kong from January 1, 2022, to December 31, 2024. Participants comprised students in Secondary 1–3 from public schools and one of their parents (the primary caregiver). Inclusion criteria for parent-child dyads are: 1) Secondary 1–3 students in public schools; 2) Both willing to participate in this study; 3) Both speak Chinese; 4) Living together; 5) The child’s main caregiver is father or mother. Exclusion criteria include: 1) Either receiving treatment for mental disorders (as the target population of the present study was the general population of adolescents and parents, and the association between depression and IGD, and its potential mediating pathways, may differ in clinical populations); 2) Either having an intellectual disability and not being able to complete the questionnaire survey independently. International schools were excluded due to significant differences in school culture, curriculum, and socioeconomic status compared to local schools. Students in Secondary 4–6 were also excluded because they need to prepare for public examinations and have a higher academic stress.

### Procedures and data collection

#### Procedures.

Stratified random sampling was used for recruitment. Out of the 18 districts in Hong Kong, eight districts were randomly selected; each selected district randomly selected one school. An invitation email was sent to selected schools, and those consented to participate would submit application forms. Subsequently, a follow-up phone call was made to discuss the details of the data collection process. Replacements of schools were made upon refusals. With the assistance of teachers and social workers, the research team distributed leaflets and consent forms to parents. To enhance response rates, the leaflets underscored the importance of parental involvement in their child’s development. As a token of appreciation, parents received supermarket e-vouchers valued at around 6.4 $, and adolescents were given bookmarks. Overall, a total of 26 schools were invited, with 12 of them agreeing to participate in the study (school-level response rate: 46.2%); at the individual level, 2,610 parent-child dyads were invited, with 1,277 dyads completing this survey (individual-level response rate: 48.9%).

#### Data collection.

Adolescents completed a self-administered, anonymous questionnaire in classrooms in the absence of teachers. Research assistants clarified that there were no “correct” or “incorrect” responses. Parents were invited to complete an online questionnaire (with a Qualtrics link) through WhatsApp during weekday evenings and weekends. If a parent did not respond after three WhatsApp reminders, follow-up phone calls were made; non-responses after three attempts of phone calls were considered invalid. As children from the same household share similar environmental and genetic factors, their responses are likely to be correlated. Therefore, a pre-specified strategy was made that parents with two or more children participating in this study would be asked to complete separate questionnaires for each child. The child’s date of birth was used to distinguish responses. For twins, the last three digits of each child’s Hong Kong ID were used for identification. However, in practice, all participating families had only one eligible child enrolled in the study. Therefore, no parent contributed to more than one parent-child dyad.

### Measurement

#### Adolescent-reported measures.

**Internet gaming disorder:** Adolescent IGD symptoms in the past year were measured using the DSM-5 IGD symptoms checklist [1], which includes nine items with total scores ranging from 0 to 9. Higher scores represent greater IGD severity, and individuals with a total score of five or more were classified as IGD cases [1]. The Chinese version of this checklist has been validated among Chinese adolescents, showing satisfactory reliability and validity [26,27]. In the present study, the Cronbach’s α was 0.75.

**Depression:** The Patient Health Questionnaire-9 (PHQ-9) [28] was used to assess adolescents’ depressive symptoms in the past two weeks. Each item was rated on a 4-point Likert scale ranging from 0 (not at all) to 3 (nearly every day), with higher total scores indicating greater symptom severity. The cutoff of five was used to indicate at least mild depressive symptoms [29]. The PHQ-9 has been validated and widely used among Chinese adolescents [29,30]. The Cronbach’s α in the present sample was 0.88.

**Gaming time:** Adolescents reported their average time spent playing Internet games on computers, consoles, tablets, or smartphones per day over the past six months. Response options include: did not play, 0.5 h/day, 1 h/day, 2 h/day, 3 h/day, 4 h/day, 5 h/day, and 6 h/day or more.

**Family relationship:** The family relationship was measured using the Relationship subscale of the 27-item Family Environment Scale [31]. This subscale assesses cohesion, expressiveness, and conflicts within a family (1 = strongly disagree to 5 = strongly agree), with the higher total scores indicating a better family relationship. It is widely used in Chinese adolescents [32]. The Cronbach’s α was 0.87 in this study.

**Background information,** including school band (ranging from 1 to 3), grade, age, gender (male/female), living arrangement (living with both parents/living with mother or father), siblings’ gaming engagement (no/yes), and mental health service history in the last year (no/yes), was collected.

#### Parent-reported measures.

**Parental IGD:** The same DSM-5 IGD symptoms checklist of adolescents was used in parents, which has been validated among adults [33]. The Cronbach’s α was 0.74 in the current parent sample.

**Depression:** To keep the parental questionnaire short, the 2-item Patient Health Questionnaire (PHQ-2) [34] was used to measure parental depressive symptoms over the past two weeks. Items are rated on a 4-point Likert scale (0 = not at all to 3 = nearly every day), with total scores ranging from 0 to 6. Participants with a total score of three or higher were considered as depression cases. The PHQ-2 has been widely used in Chinese adults [35]. The Cronbach’s α was 0.71 in this study.

**Parental gaming time:** Parental gaming time was measured using the same item for adolescents.

**Background information** includes gender, age, employment status (full-time job, part-time job, and unemployed), marital status (married or live with spouse, not married, divorced, single, and widowed), socioeconomic status (low, moderate and high), educational level (secondary school or below, high school, and college or above), and mental health service history in the past year (no and yes) was collected.

### Data matching

Parent-child data were matched using a three-step procedure. First, students were matched to parents based on school, grade, class, and date of birth. For records that remained unmatched, a second-round matching was conducted using school, grade, class, and the last three digits of the HKID number. For any participants still unmatched after the second round, the main caregiver’s phone number was used in the third step. Any remaining unmatched parent-child pairs were then manually reviewed to identify the potential errors or typos in the matching variables described above.

### Statistical analyses

Demographic characteristics of participants were presented as mean (standard deviation, SD) for continuous variables and number (percentage) for categorical variables. Pearson correlation analyses were conducted to examine correlations among the studied variables (depressive symptoms and IGD symptoms) and potential mediators (gaming time and family relationship). Following prior research [36], parent-child dyads were treated as distinguishable dyads. Little’s test (p < 0.05) provided evidence against missing completely at random. Under the missing at random/ignorable missingness assumption, the Actor-Partner Interdependence Model (APIM) was conducted using structural equation modeling with full information maximum likelihood [37]to examine interdependent associations between depressive symptoms and IGD symptoms within parent-child dyads. The Actor-Partner Interdependence Mediation Model (APIMeM) [38] was employed to test the mediating roles of gaming time and family relationship. To reduce the likelihood of overfitting, only covariates that were significant in linear mixed models (Shown in **S2** **and** **S3 Tables**) were included in the APIM and APIMeM. Age and gender of adolescents and parents were adjusted for in all models, given their established associations with IGD [2,39]. The Wald test was used to examine whether the standardized actor and partner effect of adolescents were significantly different from those of parents by constraining the corresponding standardized path coefficients to be equal, with one path tested each time. Given the non-independence of students nested within schools, following previous school-level research [40], we conducted a sensitivity analysis of APIM and APIMeM in Mplus using the function of “TYPE = COMPLEX” to adjust for the school-level cluster effect. In addition, as IGD symptoms, particularly among parents, showed a positively skewed distribution, sensitivity analyses were conducted using maximum likelihood estimation with robust standard errors (MLR) for APIM and APIMeM.

Model fit was assessed by indices of χ2/df ≤ 3, the Comparative Fit Index (CFI) > 0.90, the Tucker-Lewis Index (TLI) > 0.90, the Root Means Square Error of Approximation (RMSEA) < 0.06 and the Standardized Root Mean Square Residual (SRMR) < 0.08 [41,42]. The significance of path coefficients and 95% confidence intervals (CIs) for indirect effects was assessed using bias-corrected bootstrapping with 5,000 resamples. Standardized path coefficients and their significance were reported. The proportion of indirect effects was calculated as the ratio of the indirect effect to the total effect.

All analyses were performed using R version 4.1.3 and Mplus 7.0, and two-tailed p-values < 0.05 were considered statistically significant. The raw data used for analyses is shown in **S2 File**.

### Ethics consideration

All study procedures were conducted in accordance with the Declaration of Helsinki. Written informed consent was obtained from both adolescents and their parents. Ethical approval was granted by the Ethics Committee of the corresponding author’s institution (Reference: SBRE-19–451).

## Results

### Demographic characteristics

A total of 1277 parent-child dyads completed this survey. In adolescents (50.4% of girls), the mean age was 12.04 (SD:1.36), and most of them (83.7%) were living with both parents. Among parents, 80.8% of primary caregivers were mothers, and 41.4% had a college degree or higher (**Table 1**). The prevalence of IGD in adolescents and parents was 10.3% (132/1277) and 1.1% (14/1277), respectively. The prevalence of “at least mild depressive symptoms” was 62.8% (802/1277) in adolescents, and the prevalence of depression was 9.8% (125/1277) among parents.

**Table 1:** *Demographic characteristics of 1277 parent-child dyads*.

	Total (N = 1277 dyads), n (%)
**Adolescents**	
School band	
Band 1	791 (61.9)
Band 2	385 (30.1)
Band 3	101 (7.9)
Age (years), mean (SD)	12.04 (1.36)
≤11	494 (38.7)
12	322 (25.2)
13	275 (21.5)
≥14	184 (14.4)
Missing	2 (0.2)
Gender	
Male	629 (49.3)
Female	643 (50.4)
Missing	5 (0.4)
Living with parents	
Both parents	1069 (83.7)
Only mother/father/Neither	202 (15.8)
Missing	6 (0.5)
Mental health service history (Yes)	
No	1152 (90.2)
Yes	118 (9.2)
Missing	7 (0.5)
Sibling’s gaming engagement	
No	464 (36.3)
Yes	813 (63.7)
**Parents**	
Age (years), mean (SD)	45.36 (5.53)
≤39	148 (11.6)
40-49	821 (64.3)
≥50	308 (24.1)
Gender	
Male	245 (19.2)
Female	1032 (80.8)
Educational level	
Secondary school or below	319 (25.0)
High school	423 (33.1)
College or above	529 (41.4)
Missing	6 (0.5)
Employment status	
Full-time job	670 (52.5)
Part-time job	220 (17.2)
Unemployed	385 (30.1)
Missing	2 (0.2)
Marriage	
Married/live with spouse	1127 (88.3)
Not married/Divorced/Single/Widowed	150 (11.7)
Social economic status	
Low	644 (50.4)
Moderate	543 (42.5)
High	88 (6.9)
Missing	2 (0.2)
Mental health service history ^a^	
No	1210 (94.8)
Yes	65 (5.1)
Missing	2 (0.2)

Note: SD, standard deviation.

^a^mental health service history in the past year referred to a broader range of mental health-related service use, including both clinical services (e.g., treatment for mental disorders) and non-clinical support (e.g., counseling).

### Correlations among studied variables

Adolescent depressive symptoms were significantly correlated with both adolescent (r = 0.28, p < 0.001) and parental (r = 0.07, p < 0.05) IGD symptoms. In contrast, parental depressive symptoms was correlated only with parental IGD symptoms (r = 0.08, p < 0.01), but not with adolescent IGD symptoms. Adolescent gaming time showed positive correlations with both adolescent (r = 0.39, p < 0.001) and parental (r = 0.17, p < 0.001) IGD symptoms. Similarly, parental gaming time was positively correlated with both adolescent (r = 0.27, p < 0.001) and parental (r = 0.67, p < 0.001) IGD symptoms. Adolescent-reported family relationship was negatively correlated with depressive symptoms and IGD symptoms in both adolescents and parents (r ranging from −0.33 to −0.07, all p < 0.05) (**Table 2**).

**Table 2:** *Correlations among studied variables*.

			Adolescents				Parents	
	Score range	Mean (SD)	1	2	3	4	5	6
1. A_IGD	0-9	1.68 (1.95)	–					
2. A_Depression	0-27	7.26 (5.75)	0.28***	–				
3. A_Gaming time	0-6	1.74 (1.56)	0.39***	0.08**	–			
4. A_Family relationship	27-135	55.57 (12.39)	−0.17***	−0.33***	−0.08**	–		
5. P_IGD	0-9	0.83 (1.48)	0.39***	0.07*	0.17***	−0.08**	–	
6. P_Depression	0-6	0.91 (1.19)	0.02	0.02	−0.01	−0.07*	0.08**	–
7. P_Gaming time	0-6	0.56 (0.86)	0.27***	0.04	0.14***	−0.02	0.67***	0.02

Note: *** p < 0.001; ** p < 0.01; * p < 0.05; IGD, Internet gaming disorder; SD, standard deviation. A_ means reported by adolescents; P_ means reported by parents.

### Interdependent relationship between depressive symptoms and IGD symptoms

**Fig 1:**
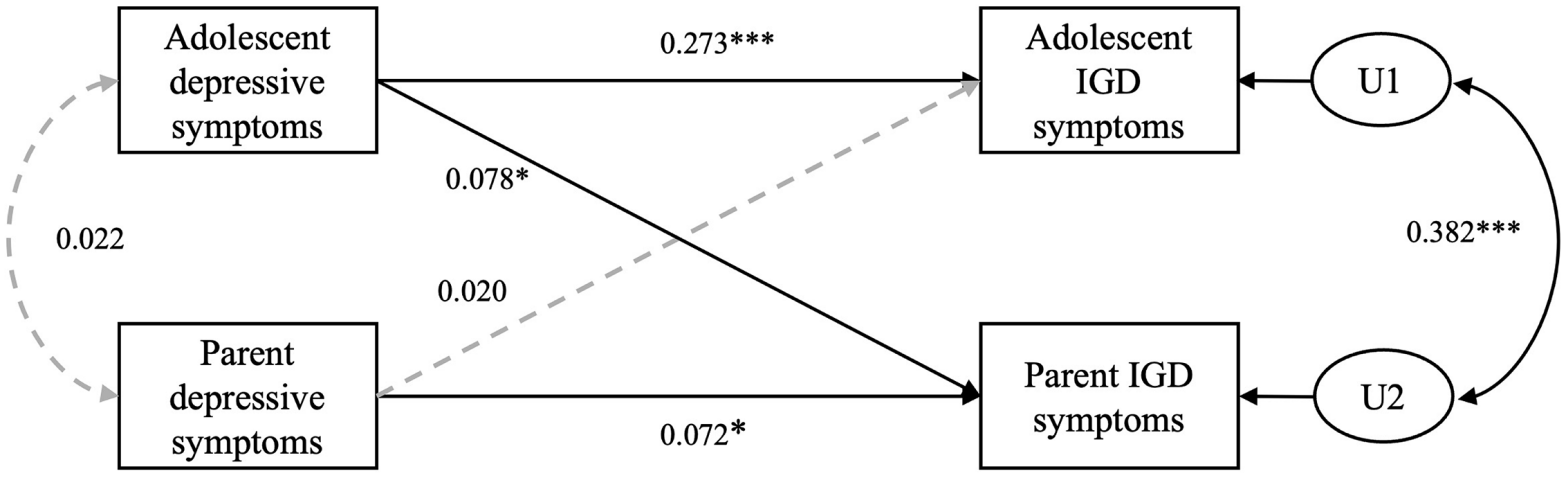
Actor-partner interdependence model. Note: standardized coefficients were shown. Age and gender of parents and adolescents were adjusted for, respectively. Solid arrows in black indicate *p* < 0.05; dashed arrows in grey indicate *p* ≥ 0.5. U1 and U2 represent residuals for adolescents’ and parental IGD symptoms; IGD, internet gaming disorder.

The tested APIM demonstrated good model fits (χ2/df = 2.789, RMSE = 0.038, SRMR = 0.023, CLI = 0.951, and TLI = 0.941; shown in **Table 3**). As shown in **Fig 1**, adolescent depressive symptoms were positively associated with their own IGD symptoms (adolescent actor effect; β = 0.273, p < 0.001) and with parental IGD symptoms (parent partner effect; β = 0.078, p < 0.05). In contrast, parental depressive symptoms were associated only with their own IGD symptoms (parent actor effect: β = 0.072, p < 0.05), but not with adolescent IGD symptoms (adolescent partner effect: β = 0.020, p > 0.05). Significant differences were found between adolescents and parents for their actor effects (0.273 vs. 0.072; Wald test p < 0.001), but not for partner effects (0.078 vs. 0.020; Wald test p = 0.177). The sensitivity analysis of adjusting the school-level cluster effect and using MLR (**S1 Fig**) showed that the significance of all estimated paths remained unchanged.

**Table 3:** *Model fits of APIM and APIMeM*.

Model fit index	APIM	APIMeM
χ2/df	2.789	2.900
RMSEA	0.038	0.039
SRMR	0.023	0.028
CFI	0.951	0.974
TLI	0.941	0.955

Note: RMSEA, Root mean square error of approximation; SRMR, Standardized root mean square residual; TLI, the Tucker-Lewis index; CFI, Comparative fit index; APIM, actor-partner interdependence model; APIMeM, actor-partner interdependence mediator model.

### Mediating roles of gaming time and family relationship

**Fig 2:**
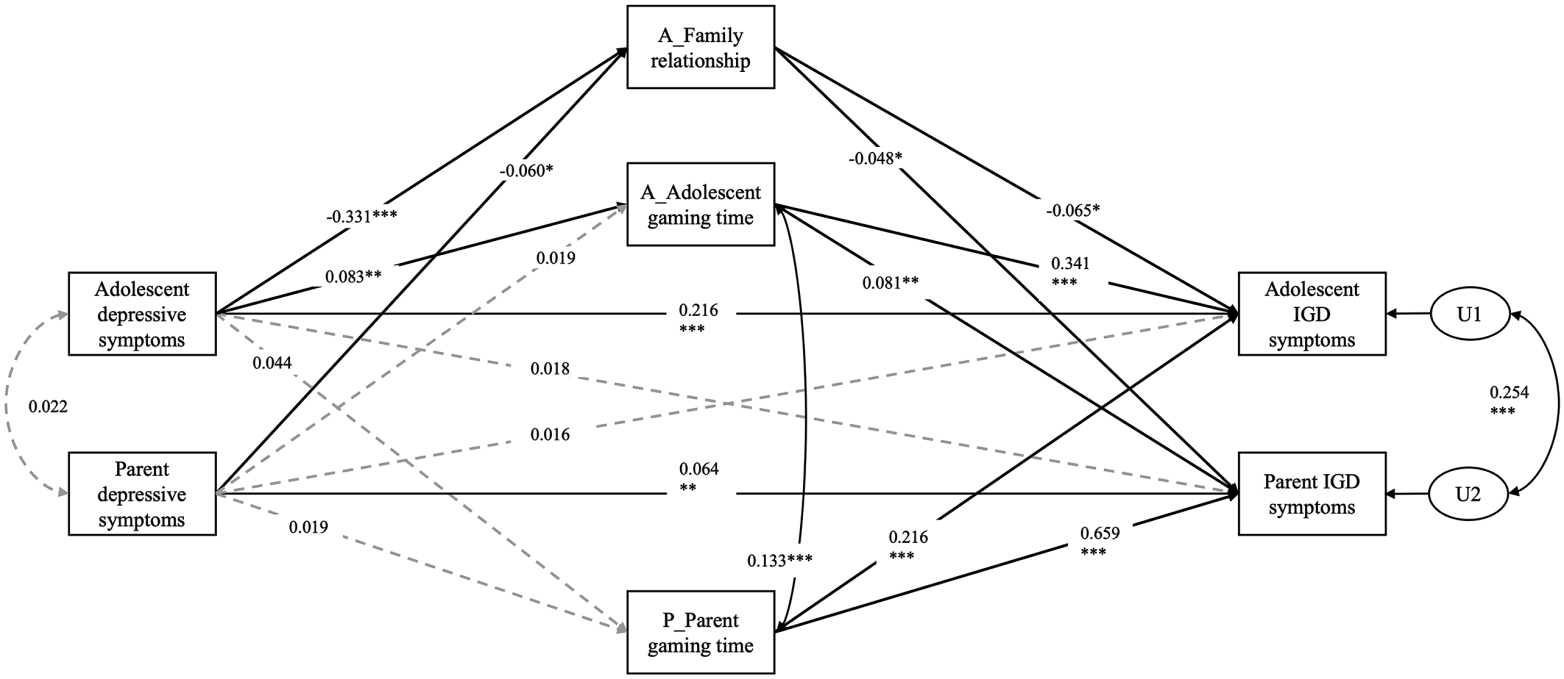
Actor-partner interdependence mediation model. Note: Standardized coefficients were shown. Age and gender of parents and adolescents were adjusted for, respectively. Solid arrows in black indicate *p* < 0.05; dashed arrows in grey indicate *p* ≥ 0.5.U1 and U2 represent residuals for adolescents’ and parental IGD symptoms; IGD, Internet gaming disorder. A_ means that the mediator was reported by adolescents; P_ means that the mediator was reported by parents.

The proposed APIMeM demonstrated good model fits (χ2/df = 2.900, RMSE = 0.039, SRMR = 0.028, CLI = 0.974, and TLI = 0.955; **Table 3**). As shown in **Fig 2**, the adolescent actor effect was partially accounted for by cross-sectional indirect associations through adolescent-reported family relationship (standardized indirect effect [95% CI] = 0.022 [0.003, 0.039]) and adolescent gaming time (0.028 [0.008, 0.051]). The total cross-sectional indirect effect accounted for 21.5% of the total effect (**Table 4**). Besides, the parent partner effect was partially accounted for by cross-sectional indirect associations through adolescent-reported family relationship (0.016 [0.001, 0.033]) and adolescent gaming time (0.007 [0.001, 0.014]), which accounted for 74.3% of the total effect. No significant cross-sectional indirect effects were observed in the associations of parental depressive symptoms with parental/adolescent IGD symptoms. The sensitivity analysis of adjusting the school-level cluster effect and using MLR (**S2 Fig**) showed that the significance of all estimated paths remained the same.

**Table 4:** *Mediation effects*.

	Effects (95%CI)	Effect/Total effect (%)	*p*
**Actor-Actor**			
Total effect	0.275 (0.219, 0.327)	–	**<0.001**
Indirect effect	0.059 (0.029, 0.090)	21.5%	**<0.001**
Direct effect	0.216 (0.164, 0.267)	78.5%	**<0.001**
**Partner-Partner**			
Total effect	0.078 (0.019, 0.137)	–	**0.009**
Indirect effect	0.014 (−0.019, 0.050)	17.9%	0.428
Direct effect	0.064 (0.021, 0.108)	82.1%	**0.004**
**Actor-Partner**			
Total effect	0.070 (0.015, 0.126)	–	**0.014**
Indirect effect	0.052 (0.012, 0.095)	74.3%	**0.014**
Direct effect	0.018 (−0.026, 0.060)	25.7%	0.409
**Partner-actor**			
Total effect	0.019 (−0.034, 0.074)	–	0.497
Indirect effect	0.003 (−0.021, 0.027)	15.8%	0.831
Direct effect	0.016 (−0.031, 0.065)	84.2%	0.504

Note: Standardized effects were shown.

## Discussions

This study is the first to examine the interdependent associations between depressive symptoms and IGD symptoms in parent-child dyads using the APIM, while also testing potential mediating mechanisms. Our findings revealed a distinct pattern of associations: significant actor effects were found for both adolescents and parents, indicating that an individual’s own depressive symptoms were a robust risk factor for their own IGD symptoms. Notably, a small but significant partner effect emerged from adolescent depressive symptoms to parental IGD symptoms, but not vice versa. This novel child-to-parent association was mediated cross-sectionally by adolescent-reported family relationship quality and adolescent gaming time.

The significant actor effect in adolescents is consistent with previous findings [43]. Notably, the actor effect was also observed in parents. It suggests that in middle-aged adults, especially in parents, the influence of emotional problems on their own digital addictions may not be overlooked. Although the prevalence of parental IGD (1.1%) was relatively low, its potential negative influences on parents’ subsequent lives may be substantial. For example, middle-aged adults with addictive behaviors were found to be associated with increased suicidal ideation [44]. These findings suggest that supporting parents in coping with negative emotions may represent potential intervention targets to prevent IGD within family. We also found that the actor effect was stronger in adolescents than in parents, indicating that adolescents may be more likely to use gaming as a maladaptive strategy to escape negative moods. Nevertheless, given that depressive symptoms were measured with different instruments in adolescents and parents, even though continuous variables were standardized, this cross-generational difference should be interpreted with caution.

A key contribution of this study is the identification of a small but significant association between adolescent depression and parental IGD, independent of parental depression and adolescent IGD. This suggests a distinct child-to-parent pathway within the family system. One possible explanation is that adolescent depression may constitute a significant psychosocial stressor for parents, potentially leading to burnout, heightened parenting stress, and family conflict [45,46]. Consistent with this, a systematic review noted that children with internalizing problems often exhibit more demanding behaviors, elevating parental stress [47]. According to the General Strain Theory [18], such stress may be related to precipitate maladaptive coping strategies. For parents, excessive gaming may serve as a form of escapism from parenting pressures, a notion supported by evidence linking avoidance coping to problematic gaming in adults [48]. This dynamic may be particularly salient in collectivist cultural contexts like Hong Kong, where a strong emphasis on familial success can intensify parental stress in response to a child’s difficulties [49]. Given the magnitude of the partner effect was small, this finding should be interpreted cautiously. As this is the first study to document this specific link, future cross-cultural, longitudinal research is needed to validate its generalizability.

Contrary to some prior studies [19,50], we did not find a significant direct partner effect from parental depression to adolescent IGD after controlling for key covariates and dyadic interdependence. This discrepancy may stem from methodological differences. Previous studies often used analytic approaches (e.g., standard regressions or SEM without dyadic controls) that could not disentangle actor effects from partner effects or fully account for shared familial factors. Our use of APIM suggests that the previously observed association may have been confounded by unmeasured dyadic similarities or the adolescents’ own depressive symptoms. This finding implies that adolescent externalizing behaviors like IGD may be more likely to be associated with observable parental behaviors, such as modeling of excessive gaming as posited by Social Learning Theory [51], rather than by parental internalizing symptoms alone. In addition, during adolescence, peer influence typically surpasses parental influence [5]. An adolescent’s decision to engage in excessive gaming is likely more strongly determined by peer norms, school stress, and personal predispositions than by their parent’s internal state of depression (though it may still be associated with parental behavior, like neglect [52]). Although the current study did not find a significant link between parental depressive symptoms and adolescent IGD, this does not imply that the potential negative effects of parental mental health issues on adolescents can be entirely overlooked. For example, parents with mental health issues often demonstrate poorer parenting practices (e.g., reduced warmth and support), which can, in turn, impose long-term detrimental effects on their child’s mental health development [53]. To have a more comprehensive understanding of adolescent behavioral dynamics, further research employing the APIM framework, is warranted to examine the similarities and differences in the depression-IGD link between friend dyads and parent-adolescent dyads.

We further found that adolescent-reported family relationship quality statistically mediated the link from adolescent depressive symptoms to both adolescent and parental IGD. Adolescent emotional distress can erode family support and increase conflict [22], prompting adolescents to seek connection in online gaming communities [54]. For parents, a strained family environment likely exacerbates stress, potentially driving them toward gaming as a coping mechanism [16] or as a misguided strategy to connect with their child by engaging in a shared activity (e.g., co-playing). Adolescent gaming time also served as a key behavioral mediator. Depressive symptoms were associated with longer gaming time, likely as an emotion-focused escape, thereby increasing IGD symptoms [55]. This excessive gaming can itself become a source of parental concern and stress, potentially prompting parents to engage in gaming as a shared activity or a maladaptive coping strategy, thereby increasing their own IGD symptoms. While parental gaming time was not a mediator in our model, it was significantly associated with IGD symptoms for both children and parents, highlighting it as a potential critical role in preventing IGD. In addition, although gender may be a potential moderator of the association between depressive symptoms and IGD, we did not examine whether the dyadic associations and mediation pathways differed by adolescent and parent gender. This was because the parental sample was highly gender-imbalanced with over 80% being mothers, and the gender-stratified sample sizes (2 genders of adolescents * 2 genders of parents) would have been too small to conduct these dyadic analyses, especially for the APIMeM. Future studies with larger and more gender-balanced parental samples are needed to examine potential gender differences in these dyadic associations and mediation pathways.

### Implications

Theoretically, this study advances the field by applying a dyadic framework to the depression-IGD link. It confirms depression as a transdiagnostic risk factor at the individual level while uncovering a novel, child-driven pathway within the family system, challenging purely parent-effect models. Practically, our findings suggest that family-system and transdiagnostic interventions may represent useful directions to consider. First, interventions may target communication and emotional support within the family. Schools and community centers could offer parenting workshops focused on adolescent mental health, while governments could improve access to family counseling. Second, clinicians may consider assessing depression and gaming behaviors in both adolescents and parents, as these issues are interlinked. Last but not least, families may be supported in establishing clear, age-appropriate guidelines for gaming. Encouraging shared offline activities may provide alternative bonding experiences. The development of evidence-based, practical gaming time guidelines for different age groups is a crucial need for future research.

### Limitations

Several limitations should be considered when interpreting these findings. First, the cross-sectional design precludes definitive causal inference. It is possible that IGD symptoms may in turn increase the risk of depression. Future longitudinal research is essential to establish the temporal and potentially reciprocal relationships between depressive symptoms and IGD within dyads. Second, our measure of family relationship quality relied solely on adolescent reports, which might only reflect adolescents’ mood and subjective perceptions instead of the dyadic family functioning. This single-informant approach did not capture parents’ perspective. Therefore, the mediating role of family relationships should be interpreted cautiously. Third, the adolescent sample was recruited exclusively from school settings. Adolescents who had dropped out of school or were absent on the survey day were excluded. This may limit the generalizability of our findings, particularly to more vulnerable adolescents who are not currently attending school. However, the rate of adolescents at school age but not enrolling in school is low, ranging from 0.8% to 3.5% in ten years since 2014 [56]. Therefore, its influence on our results should be small. Future studies should consider broader sampling strategies to include a more representative adolescent sample. Fourth, the observed actor effect in adolescents and cross-sectional mediating effects of adolescent-reported family relationships and adolescent gaming time may be inflated by common-method bias, as these variables were all self-reported by adolescents through questionnaires. Future studies are advised to adopt multi-informant designs (e.g., objective gaming logs) to validate these findings. Fifth, the response rate was low (48.9%), and parent-child dyads who agreed to participate may differ from nonparticipants (e.g., families with better family functioning may be more likely to participate). This may lead to selection bias and limit the generalizability of the findings. Last but not least, depressive symptoms were measured using different instruments in parents (PHQ-2) and adolescents (PHQ-9). Although the variables were standardized before analysis, this asymmetry in measurement depth and sensitivity may limit the comparability of actor and partner effects across generations. The findings regarding cross-generational differences should still be interpreted cautiously.

## Conclusions

Adolescent depressive symptoms were positively associated with both their own and parents’ IGD symptoms, with adolescent-reported family relationships and adolescent gaming time serving as mediators. The close association of adolescents’ mental health problems with parents’ problematic behaviors should not be overlooked. Family-based, transdiagnostic interventions targeting both depression and IGD may represent promising prevention and intervention directions for reducing IGD across the generations.

## Supporting information

S1 File Details of the search strategy. (DOCX)

S2 File Raw dataset. (CSV)

S1 Table Literature review of studies on the relationship between parental modeling and adolescent IGD. (DOCX)

S2 Table Risk factors of parental IGD. (DOCX)

S3 Table Risk factors of adolescent IGD. (DOCX)

S1 Fig Actor-partner interdependence model by using maximum likelihood estimation with robust standard errors. (DOCX)

S2 Fig Actor-partner interdependence mediation model by using maximum likelihood estimation with robust standard errors. (DOCX)
