# Supplementary material for: Interdependent relationship between depression and Internet gaming disorder in parent-child dyads: The mediating role of family relationship and gaming time
Source: PLoS One. 2026 Jun 15;21(6):e0351947. doi: 10.1371/journal.pone.0351947 (PMC13268149; doi:10.1371/journal.pone.0351947)
Supplement: S1 File — (DOCX) [file pone.0351947.s001.docx]

**S1. Details of the search strategy**

## **1.1. Literature review on parental IGD and adolescent IGD**

Preliminary search results (November 11, 2025)

| **Database** | N of studies |
| --- | --- |
| MEDLINE | 45 |
| Embase | 139 |
| Web of Science | 144 |
| PsycINFO | 49 |
| **Deduplicated** | 128 |
| **Included** | 247 |

**Ovid MEDLINE**(R) and Epub Ahead of Print, In-Process, In-Data-Review & Other Non-Indexed Citations

| **#** | **Query** | **Results** |
| --- | --- | --- |
| 1 | Parents/ or Mothers/ or Fathers/ or Caregivers/ or Legal Guardians/ | 213,256 |
| 2 | (parent* or mother* or father* or caregiver* or paternal or maternal or guardian).ab,ti. | 974,350 |
| 3 | Child/ or Adolescent/ or Students/ | 3,400,147 |
| 4 | (child* or adolescent* or youth or student* or teen* or juvenile or offspring).ab,ti. | 2,207,990 |
| 5 | ((gaming or game*) adj5 (addict* or dependen* or overuse or abuse or disorder* or excessive or misuse or pathology* or problem* or compulsive)).ab,ti. | 2,953 |
| 6 | Depression/ or Depressive Disorder/ or Depressive Disorder, Major/ or Mood Disorders/ | 291,796 |
| 7 | (depress* or MDD or dysthymi or "affective disorder" or "affective disorders" or "mood disorder" or "mood disorders").ab,ti. | 564,869 |
| 8 | 1 or 2 | 1,010,455 |
| 9 | 3 or 4 | 4,190,122 |
| 10 | 6 or 7 | 612,270 |
| 11 | 5 and 8 and 9 and 10 | 45 |
| 12 | limit 11 to humans | 45 |

**Embase**

| **#** | **Query** | **Results** |
| --- | --- | --- |
| 1 | parent/ or mother/ or father/ or caregiver/ | 377,255 |
| 2 | (parent* or mother* or father* or caregiver* or paternal or maternal or guardian).ab,ti. | 1,505,037 |
| 3 | child/ or adolescent/ or juvenile/ or elementary student/ or high school student/ or student/ | 3,609,077 |
| 4 | (child* or adolescent* or youth or student* or teen* or juvenile or offspring).ab,ti. | 3,345,514 |
| 5 | game addiction/ | 3,284 |
| 6 | ((gaming or game*) adj5 (addict* or dependen* or overuse or abuse or disorder* or excessive or misuse or pathology* or problem* or compulsive)).ab,ti. | 5,670 |
| 7 | depression/ or major depression/ or depressive psychosis/ or mood disorder/ | 717,887 |
| 8 | (depress* or MDD or dysthymi or "affective disorder" or "affective disorders" or "mood disorder" or "mood disorders").ab,ti. | 917,749 |
| 9 | 1 or 2 | 1,561,663 |
| 10 | 3 or 4 | 4,922,393 |
| 11 | 5 or 6 | 6,192 |
| 12 | 7 or 8 | 1,116,057 |
| 13 | 9 and 10 and 11 and 12 | 145 |
| 14 | limit 13 to human | 139 |

**Web of Science Core Collection**

| **#** | **Search Query** | **Results** |
| --- | --- | --- |
| 1 | TS=(parent* OR mother* OR father* OR caregiver* OR paternal OR maternal OR guardian) | 1679489 |
| 2 | TS=(child* or adolescent* or youth or student* or teen* or juvenile or offspring) | 4678053 |
| 3 | TS = ((gaming OR game*) NEAR/5 (addict* or dependen* or overuse or abuse or disorder* or excessive or misuse or pathology* or problem* or compulsive)) | 21313 |
| 4 | TS = (depress* OR MDD OR dysthymi OR "affective disorder" OR "affective disorders" OR "mood disorder" OR "mood disorders") | 1033780 |
| 5 | #1 AND #2 AND #3 AND #4 | 144 |

**PsycInfo**

| **#** | **Query** | **Results** |
| --- | --- | --- |
| 1 | Parents/ or Mothers/ or Fathers/ or Caregivers/ | 151,296 |
| 2 | (parent* or mother* or father* or caregiver* or paternal or maternal or guardian).ab,ti. | 516,729 |
| 3 | Elementary School Students/ or High School Students/ or Junior High School Students/ or Students/ or Offspring/ | 130,461 |
| 4 | (child* or adolescent* or youth or student* or teen* or juvenile or offspring).ab,ti. | 1,602,417 |
| 5 | ((gaming or game*) adj5 (addict* or dependen* or overuse or abuse or disorder* or excessive or misuse or pathology* or problem* or compulsive)).ab,ti. | 4,276 |
| 6 | Major Depression/ or "Depression (Emotion)"/ or Affective Disorders/ or Affective Disorders/ | 213,929 |
| 7 | (depress* or MDD or dysthymi or "affective disorder" or "affective disorders" or "mood disorder" or "mood disorders").ab,ti. | 410,182 |
| 8 | 1 or 2 | 527,179 |
| 9 | 3 or 4 | 1,619,848 |
| 10 | 6 or 7 | 420,392 |
| 11 | 5 and 8 and 9 and 10 | 49 |
| 12 | limit 11 to human | 47 |
