# Supplementary material for: Interdependent relationship between depression and Internet gaming disorder in parent-child dyads: The mediating role of family relationship and gaming time
Source: PLoS One. 2026 Jun 15;21(6):e0351947. doi: 10.1371/journal.pone.0351947 (PMC13268149; doi:10.1371/journal.pone.0351947)
Supplement: S1 Table — (DOCX) [file pone.0351947.s003.docx]

## **S1 Table. Literature review of studies on the relationship between parental modeling and adolescent IGD**

| Study | Study design | Sample size | Analyses | Covariates adjusted for | Results |
| --- | --- | --- | --- | --- | --- |
| Wartberg, 2017, Germany[1] | Cross-sectional | 1095 parent-child dyads (85.5% of mothers) | Logistic regression | Adolescent's gender, antisocial behavior, anger control problems, emotional distress, self-esteem problems, hyperactivity/inattention; parent's gender | A lower parental depression was associated with adolescent IGD |
| Wartberg, 2018, Germany[2] | Longitudinal | 985 parent-child dyads (85.8% of mothers) | SEM | None | No significant associations were found |
| Jeong, 2021, South Korea[3] | Cohort | 2031 parent-child dyads (93.4% of mothers) | GENMOD procedure to estimate RR | Adolescent's age, gender, socioeconomic status, and family type, and time spent playing online game during weekdays | No significant associations were found |
| Piao, 2022, South Korea[4] | Longitudinal | 778 adolescents and 685 parents (92.4% of mothers) | SEM | None | Parents’ depression at T1 predicted adolescents' pathological gaming at T3 through adolescents' aggression, ADHD and self-control at T2 |
| Lam, 2022, China[5] | Cross-sectional | 104 parent-child dyads (58.7% of mothers) | Hierarchical logistic regression | Child's age, gender, gaming activity engagement, other screen time activity engagement, sport activity engagement; parent's age, gender, education | Parental depressive symptoms was positively associated with cihldren's IGD symptoms |
| Lin, 2023, China[6] | Cross-sectional | 4385 parent-child dyads (69.8% of mothers) | SEM | None | Parent-reported parental depression was related to worse adolescent-reported parent-child relationship, which in turn related to more severe adolescent-reported problematic gaming |

Note: IGD, Internet gaming disorder; SEM, structural equation modeling.

**References**

1. Wartberg, L., et al., *Internet gaming disorder in early adolescence: Associations with parental and adolescent mental health.* European Psychiatry, 2017. **43**: p. 14-18.

2. Wartberg, L., et al., *A longitudinal study on psychosocial causes and consequences of Internet gaming disorder in adolescence.* Psychological medicine, 2019. **49**(2): p. 287-294.

3. Jeong, H., et al., *Joint effects of children’s emotional problems and parental depressive symptoms on the occurrence of internet gaming disorder among children and adolescents: A longitudinal study.* Journal of Behavioral Addictions, 2021. **10**(2): p. 244-252.

4. Piao, M.Y., E.J. Jeong, and J.A. Kim. *Mental health of parents and their children: A longitudinal study of the effects of parents’ negative affect on adolescents’ pathological gaming*. in *Healthcare*. 2022. MDPI.

5. Lam, Y.-T. and C. Cheng, *Parental depression and leisure activity engagement on children’s gaming disorder: A dyadic study.* International Journal of Environmental Research and Public Health, 2022. **19**(10): p. 5880.

6. Lin, L., R. Ding, and S. Ni, *How does parents' psychological distress relate to adolescents' problematic gaming? The roles of parent-adolescent relationship and adolescents' emotion regulation.* Journal of Behavioral Addictions, 2023. **12**(4): p. 953-963.
