# Supplementary material for: Interdependent relationship between depression and Internet gaming disorder in parent-child dyads: The mediating role of family relationship and gaming time
Source: PLoS One. 2026 Jun 15;21(6):e0351947. doi: 10.1371/journal.pone.0351947 (PMC13268149; doi:10.1371/journal.pone.0351947)
Supplement: S2 Table — (DOCX) [file pone.0351947.s004.docx]

## **S2 Table. Risk factors of parental IGD**

|  | Model 1 |  | Model 2 |  |
| --- | --- | --- | --- | --- |
|  | b (95%CI) | *p* | b (95%CI) | *p* |
| **Adolescents' factors** |  |  |  |  |
| Depression | 0.016 (0.003, 0.028) | **0.014** | 0.019 (0.006, 0.031) | **0.004** |
| School band |  |  |  |  |
| Band 1 | Ref. | **-** | **-** | **-** |
| Band 2 | 0.079 (-0.213, 0.372) | 0.611 | **-** | **-** |
| Band 3 | 0.270 (-0.203, 0.743) | 0.299 |  |  |
| Age | -0.067 (-0.129, -0.004) | **0.038** | -0.055 (-0.115, 0.006) | 0.080 |
| Gender |  |  |  |  |
| Male | Ref. | **-** | Ref. | **-** |
| Female | -0.272 (-0.424, -0.119) | **0.001** | -0.297 (-0.449, -0.146) | **<0.001** |
| Living with parents |  |  |  |  |
| Both parents | Ref. | **-** | **-** | **-** |
| Only mother/father/Neither | -0.181 (-0.379, 0.017) | 0.073 | **-** | **-** |
| Sibling's gaming engagement |  |  |  |  |
| No | Ref. | **-** | **-** | **-** |
| Yes | 0.123 (-0.111, 0.357) | 0.303 | **-** | **-** |
| Mental health service history |  |  |  |  |
| No | Ref. | **-** | Ref. | **-** |
| Yes | 0.258 (0.004, 0.512) | **0.047** | 0.197 (-0.060, 0.454) | 0.133 |
| **Parental factors** |  |  |  |  |
| Depression | 0.088 (0.028, 0.148) | **0.004** | 0.081 (0.022, 0.140) | **0.007** |
| Age | -0.010 (-0.022, 0.003) | 0.130 | -0.018 (-0.031, -0.005) | **0.007** |
| Gender |  |  |  |  |
| Male | Ref. | **-** | Ref. | **-** |
| Female | -0.296 (-0.472, -0.120) | **0.001** | -0.362 (-0.545, -0.179) | **<0.001** |
| Educational level |  |  |  |  |
| Secondary school or below |  |  |  |  |
| High school | 0.124 (-0.078, 0.327) | 0.230 | **-** | **-** |
| College or above | 0.061 (-0.141, 0.263) | 0.554 | **-** | **-** |
| Employment status |  |  |  |  |
| Full-time job |  |  |  |  |
| Part-time job | -0.125 (-0.337, 0.087) | 0.248 | **-** | **-** |
| Unemployed | -0.115 (-0.276, 0.047) | 0.164 | **-** | **-** |
| Marriage |  |  |  |  |
| Married/live with spouse | Ref. | **-** | **-** | **-** |
| Not married/Divorced/Single/Widowed | -0.030 (-0.250, 0.190) | 0.792 | **-** | **-** |
| Social economic status |  |  |  |  |
| Low | Ref. | **-** | **-** | **-** |
| Moderate | -0.026 (-0.177, 0.125) | 0.738 | **-** | **-** |
| High | -0.015 (-0.294, 0.263) | 0.914 | **-** | **-** |
| Mental health service history |  |  |  |  |
| No | Ref. | **-** | **-** | **-** |
| Yes | 0.014 (-0.310, 0.338) | 0.932 | **-** | **-** |

Note: Model1 is univariable analysis; Model2 is multivariable analysis. All models adjusted for cluster effects within schools.

b, unstandardized coefficients of linear mixed models.
