# Supplementary material for: Interdependent relationship between depression and Internet gaming disorder in parent-child dyads: The mediating role of family relationship and gaming time
Source: PLoS One. 2026 Jun 15;21(6):e0351947. doi: 10.1371/journal.pone.0351947 (PMC13268149; doi:10.1371/journal.pone.0351947)
Supplement: S3 Table — (DOCX) [file pone.0351947.s005.docx]

## **S3 Table. Risk factors of adolescent IGD**

|  | Model 1 |  | Model 2 |  |
| --- | --- | --- | --- | --- |
|  | β (95%CI) | *p* | β (95%CI) | *p* |
| **Adolescents' factors** |  |  |  |  |
| Depression | 0.093 (0.075, 0.111) | **<0.001** | 0.101 (0.082, 0.120) | **<0.001** |
| School band |  |  |  |  |
| Band 1 | Ref. | **-** | **-** | **-** |
| Band 2 | 0.220 (-0.249, 0.689) | 0.392 | **-** | **-** |
| Band 3 | -0.668 (-1.426, 0.090) | 0.131 | **-** | **-** |
| Age | 0.009 (-0.091, 0.109) | 0.861 | 0.004 (-0.092, 0.100) | 0.941 |
| Gender |  |  |  |  |
| Male | Ref. | **-** | Ref. | **-** |
| Female | -0.590 (-0.823, -0.356) | **<0.001** | -0.784 (-1.010, -0.558) | **<0.001** |
| Living with parents |  |  |  |  |
| Both parents | Ref. | **-** | **-** | **-** |
| Only mother/father/Neither | 0.070 (-0.230, 0.371) | 0.647 | **-** | **-** |
| Sibling's gaming engagement |  |  |  |  |
| No | Ref. | - | - | - |
| Yes | 0.087 (-0.066, 0.240) | 0.265 | - | - |
| Mental health service history |  |  |  |  |
| No | Ref. | **-** | Ref. | **-** |
| Yes | 0.548 (0.164, 0.933) | **0.005** | 0.166 (-0.208, 0.541) | 0.384 |
| **Parental factors** |  |  |  |  |
| Depression | 0.046 (-0.044, 0.137) | 0.315 | **-** | **-** |
| Age | -0.013 (-0.032, 0.006) | 0.173 | -0.021 (-0.040, -0.002) | **0.029** |
| Gender |  |  |  |  |
| Male | Ref. | **-** | Ref. | **-** |
| Female | -0.076 (-0.343, 0.192) | 0.579 | -0.149 (-0.415, 0.117) | 0.272 |
| Educational level |  |  |  |  |
| Secondary school or below | Ref. | - | **-** | **-** |
| High school | 0.109 (-0.201, 0.418) | 0.492 | **-** | **-** |
| College or above | 0.007 (-0.303, 0.316) | 0.965 | **-** | **-** |
| Employment status |  |  |  |  |
| Full-time job | Ref. | **-** | **-** | **-** |
| Part-time job | 0.050 (-0.271, 0.372) | 0.758 | **-** | **-** |
| Unemployed | -0.029 (-0.274, 0.216) | 0.817 | **-** | **-** |
| Marriage |  |  |  |  |
| Married/live with spouse | Ref. | **-** | **-** | **-** |
| Not married/Divorced/Single/Widowed | 0.198 (-0.135, 0.530) | 0.245 | **-** | **-** |
| Social economic status |  |  |  |  |
| Low | Ref. | **-** | **-** | **-** |
| Moderate | -0.040 (-0.269, 0.189) | 0.734 | **-** | **-** |
| High | -0.205 (-0.627, 0.217) | 0.340 | **-** | **-** |
| Mental health service history |  |  |  |  |
| No | Ref. | **-** | **-** | **-** |
| Yes | 0.301 (-0.189, 0.791) | 0.229 | **-** | **-** |

Note: Model1 is univariable analysis; Model2 is multivariable analysis. All models adjusted for cluster effects within schools.

b, unstandardized coefficients of linear mixed models.
