# Supplementary material for: Interdependent relationship between depression and Internet gaming disorder in parent-child dyads: The mediating role of family relationship and gaming time
Source: PLoS One. 2026 Jun 15;21(6):e0351947. doi: 10.1371/journal.pone.0351947 (PMC13268149; doi:10.1371/journal.pone.0351947)
Supplement: S1 Fig — (DOCX) [file pone.0351947.s006.docx]

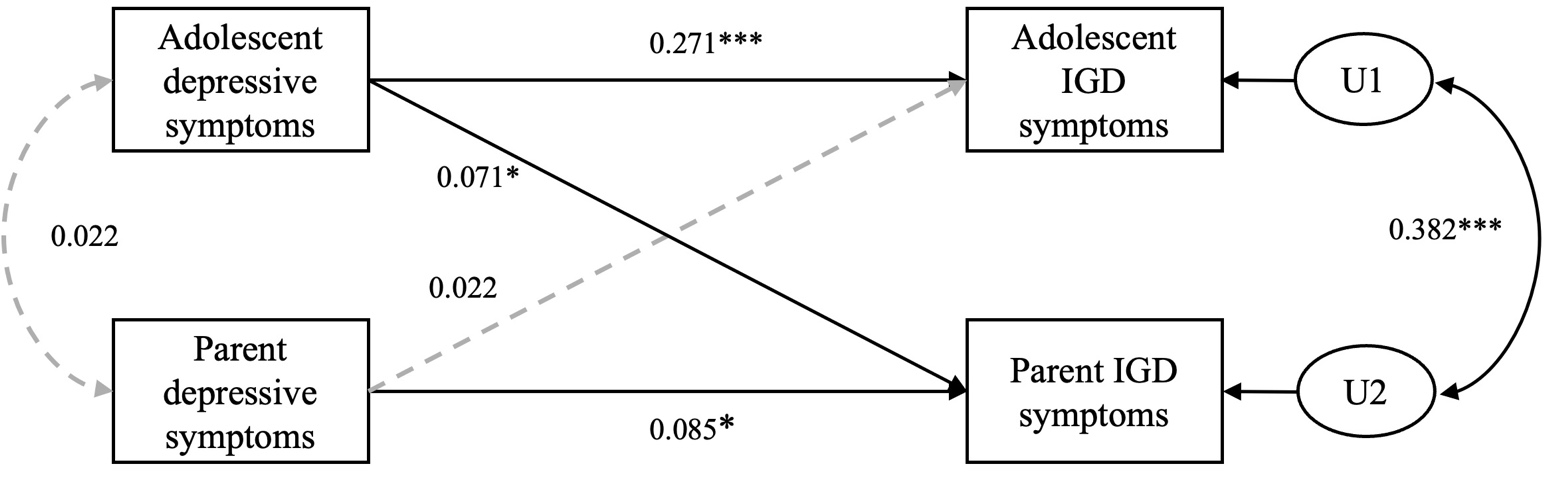


**S1 Figure. Actor-partner interdependence model by using maximum likelihood estimation with robust standard errors**

Note: Standardized coefficients were shown. Age and gender of parents and adolescents were adjusted for, respectively. Solid arrows in black indicate p < 0.05; dashed arrows in grey indicate p ≥ 0.5.U1 and U2 represent residuals for adolescents’ and parental IGD symptoms; IGD, Internet gaming disorder. ***p<0.001; ** p<0.01; * p<0.05
